# How Can CpG Methylations, and Pair-to-Pair Correlations between the Main (Gene) and the Opposite Strands, Suggest a Bending DNA Loop: Insights into the 5′-UTR of DAT1

**DOI:** 10.3390/genes14010190

**Published:** 2023-01-11

**Authors:** Vincenza Di Paola, Martina Morrone, Valentina Poli, Andrea Fuso, Esterina Pascale, Walter Adriani

**Affiliations:** 1 Faculty of Psychology, Uninettuno University, 00186 Rome, Italy; 2 Department of Experimental Medicine, “Sapienza” University of Rome, 00161 Rome, Italy; 3 Department of Medical-Surgical Sciences and of Biotechnologies, “Sapienza” University of Rome, 00161 Rome, Italy; 4 Center for Behavioral Sciences and Mental Health, Istituto Superiore di Sanità, 00161 Rome, Italy

**Keywords:** methylation, DNA secondary structure, regulatory promoter region, gene expression, double-helix opening

## Abstract

A working hypothesis issues from patterns of methylation in the 5′-UTR of the DAT1 gene. We considered relationships between pairs of CpGs, of which one on the main-gene strand and another on the complementary opposite strand (COS). We elaborated on data from ADHD children: we calculated all possible combinations of probabilities (estimated by multiplying two raw values of methylation) in pairs of CpGs from either strand. We analyzed all correlations between any given pair and all other pairs. For pairs correlating with M6-M6COS, some pairs had cytosines positioning to the reciprocal right (e.g., M3-M2COS and M6-M5COS), other pairs had cytosines positioning to the reciprocal left (e.g., M2-M3COS; M5-M6COS). Significant pair-to-pair correlations emerged between main-strand and COS CpG pairs. Through graphic representations, we hypothesized that DNA folded to looping conformations: the C^1^GG C^2^GG C^3^GG and C^5^G C^6^G motifs would become close enough to allow cytosines 1-2-3 to interact with cytosines 5-6 (on both strands). Data further suggest a sliding, with left- and right-ward oscillations of DNA strands. While thorough empirical verification is needed, we hypothesize simultaneous methylation of main-strand and COS DNA (“methylation dynamics”) to serve as a promising biomarker.

## 1. Introduction

The methylation of DNA occurs mainly at the cytosine-phosphate-guanine (CpG) sites, with the addition of a methyl group (-CH3) at position C5 (fifth carbon) of the cytosine, to form 5-methyl-cytosine (5-mC). As discussed earlier [1], each CpG on one DNA strand faces a CpG on the opposite DNA strand. The result after modifying such cytosine residues could be that two methylated cytosines are positioned diagonally on opposite DNA strands. Almost exclusively in motifs, i.e. with more than just one 5′-CpG-3′ in a row, such as in …CGCG… or …CGGCGG… sequences, each second cytosine will face, diagonally, the two cytosines that are complementary to both the preceding and the following guanines [1]. As such, multiple interactions among cytosines may become likely. 

Given this, we had the innovative idea to estimate the likelihood that all these cytosines, residing in such motifs, might be all methylated (or not) simultaneously [2,3,4,5]. In order to introduce this basic idea, note that an estimate of the *simultaneous* DNA methylation can be served by multiplying the raw values of (de)methylation in pairs of CpGs present in the DNA strand. Additionally, we proposed a crucial role for the “matrix-table” calculating correlations among (pairs of) loci. With Pearson’s R, our study was the first ever to analyze all correlations between all possible combinations of probability, estimated through multiplications [5].

The present study is a theoretical development starting from evidence gathered from our recent study [4]. In contrast to all previous epigenetic studies, in which only the gene strand is usually evaluated (also in [1,6]), the recent work of Lambacher et al. ([4]) considered all existing correlations between all pairs, one in the main (gene) strand and one in the *complementary opposite strand* (COS).

The main functional implication (reported in that study [4]) was an unexpected dependency relationship between two positions, i.e., C-p-G 1 on the gene strand could be methylated if C-p-G 6 on COS was also methylated. To our knowledge, such kind of dependency has never been reported before for any DNA motif. At least for the 5′-UTR of the DAT1 gene, interactions exist between the first CpG of the first motif on the gene strand and the first CpG of the second motif on the antiparallel COS strand.

Out of all the analyses we had run, we focused only on the situations with methylation of the gene strand and de-methylation of the opposite strand (MxDy-cos) or, conversely, de-methylation of the gene strand and methylation of the opposite strand (DxMy-cos). For the present study, therefore, we considered all correlations between all pairs in which the situation was now of methylation on both strands (MxMy-cos). We then propose an inventive interpretation of what kind of insight this approach may provide. To follow our reasoning, we underline that a multiplication of probabilities is a classical index for simultaneous events.

## 2. Methods

Between 2010 and 2012, trained child neuropsychiatrists (ADHD Clinical Unit, led by prof. Paolo Curatolo, Tor Vergata University of Rome, Italy) recruited school-aged children (6–12 years old), diagnosed according to DSM-IV and ICD-10 criteria plus Conners’ scales and k-SADS. This study was approved by the Ethical Committee of ISS; rules set by the Code of Ethics of the World Medical Association (“Declaration of Helsinki”), as printed in the British Medical Journal (18 July 1964), were fully respected.

### 2.1. Assessment of Methylation Both on Gene-Main Strand and on COS

Out of the originally recruited patients, we selected a cohort of 14 ADHD patients (half 9\10 and half 10\10 genotype), for which we also assessed the other strand (see [6]), between 2016 and 2017. Samples were processed to assess the pattern of methylation within the 5′-untranslated region (5′-UTR) of the DAT1 gene, as well as their COS on the non-coding strand: six specific CpG residues termed M1, M2, M3, M5, M6, and M7 are found after the transcriptional start site [2,3,6]. M1 to M7 CpG residues are underscored in the following sequence, being ordered from left to right: +714 **CG**G **CG**G **CG**G CTT GCC GGA GAC T**CG CG**A GCT C**CG** +746 (first intron). Notably, the CpG residues that would be named M4 (+728 and +729) shall be discarded, because of a SNP which may eliminate this whole CpG in some individuals. M1–M3 (+714-715, +717-718, +720-721) represent a CGGCGGCGG motif, while contiguous M5-M6 (+736-737 and +738-739) represents a CGCG motif. The same DNAs of patients were processed again, to assess the region corresponding to DAT1 5′-UTR both on the coding and the non-coding strands. The following primers (5′–3′) were used for amplification: on the main-gene strand, forward 5′-AGCTACCATG CCCTATGTGG-3′—reverse 5′-ATCAGCACTC CAAACCCAAC-3′; on the other strand, h_SLC6A3_Rev PyroMark Custom Assay—forward 5′-AGGTGGAGGT TTTAATAGGTAAT-3′—reverse [Biotin] 5′-AACCACATTT TACTATATAAACCCA-3′—All the methodological pyro-sequencing details are same as provided in the original study [6].

**Figure 1:**
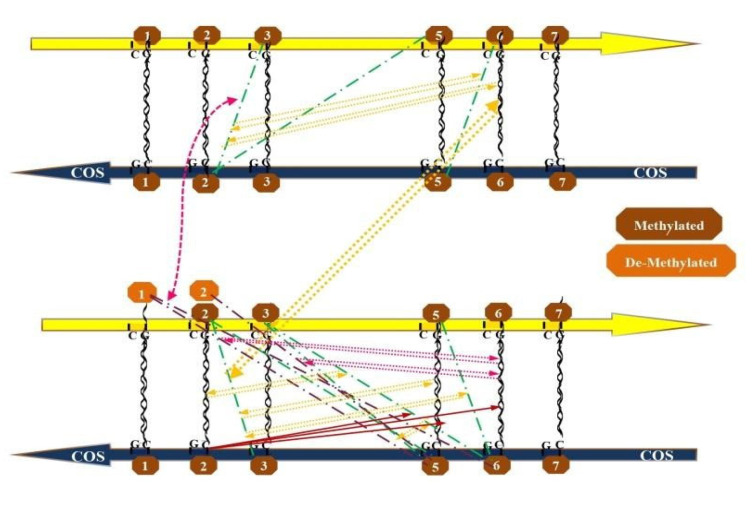
Graphic representation of significant recurrent interactions between certain methylated and sometimes de-methylated CpG pairs, accounting for a tight interaction between cytosines placed one another at their right and giving clues for a leftward slip between the strands (see Figure 4). Each methylated pair is represented by the green point-line-point hatching, the correlations between pairs are drawn by the two-way dashed arrows in yellow. Some main-strand de-methylated\COS-methylated pairs are represented by the purple point-line-point hatching, the correlations between such pairs and the M6-M6COS pair are drawn by the two-way dashed arrows in purple.

To sum up, the 5′-UTR region of the dopamine transporter (DAT1) gene (*SLC6A3*) has two nearby +714 CGG CGG CGG +722 and +736 CGCG +739 motifs, located at the first intron, downward from TSS and before the AUG, in the portion of the gene that is transcribed, spliced out from the mRNA: i.e., its beginning portion which is not yet translated (see [1], Figure 1).

### 2.2. Search for Recurrent Interactions between (de)Methylated CpG Pairs

As a first step, all raw methylation levels were multiplied so that in each pair one main-gene CpG was multiplied with one COS: we set the combinatory scheme to list all pairs, starting from M1-M1COS, M1-M2COS, M1-M3COS, … M2-M1COS, M2-M2COS, … up to M7-M7COS (a total of 6 times 6 = 36 pairs). As an internal control, we included CpG 7 (not part of motifs).

We then prepared the tables calculating the correlations between a given pair and all the other 35 pairs, realizing a matrix of 1296 cells (of which 36 cells had the value of 1 when both members of the multiplied pairs were identical, e.g., M1-M1COS correlating with M1-M1COS). Of course, we did not take into account correlations where the same locus appeared in both correlation terms: for instance, the Pearson’s R between M2-M1COS and M2-M3COS was not considered (as CpG 2 recurs twice). Hence, each line of the table did not consider a further ten values (marked with “\\” in Table 1); still, correlations were run between a given pair and all the other 25 pairs. Thus, we ended up with a total of 36 times 25 = 900 cells. Now, such a big table would be quite hard to follow; therefore, we are hereby presenting the most noteworthy findings.

## 3. Results

Table 1 provides an extract of the whole matrix: just to give an example of how data look like, we chose to show interactions between CpG 1 (in pair with all COS CpGs) and CpGs 2 or 6 (in pair with all COS CpGs). This choice is justified by the opposite roles of CpG 1 vs. CpGs 2 or 6 in the prognosis of ADHD [2,3,6]. The majority of the cells yielded quite low correlations, yet some specific cells return a significant R-value (R > 0.56). In Table 1, for instance, only two cells trespass this threshold, these being M1-M6COS with M2-M1COS (R = 0.6889) and M1-M5COS with M2-M2COS (R = 0.5682). The first notion from these results is that CpG M1, interacting with either CpG M5COS or M6COS, gets methylated along with CpG M2, interacting with either CpG M2COS or M1COS; the picture is quite specific, however, since the CpGs M5COS and M2COS are involved preferentially together, and so are CpGs M6COS and M1COS. The other possible combinations, i.e., interactions of M1 paired to M1COS or M2COS with M2 paired to M5COS or M6COS, yield very low R-values (R < 0.2837 but see all six cells with underlined R-values in Table 1). Therefore, a pair of CpGs (one in either strand) gets methylated preferentially with another specific pair of CpGs.

To fully interpret the implications of such a picture, more details on the patterns of significant cells emerging from our approach are needed. When a full list of significant correlations with all the other pairs was drawn, we noticed that only a few of these cells (each one involving one pair with another pair) had high Pearson’s R-values. In this way, the full network of related pairs was drawn. Interestingly, this list could be easily divided into two parts, based on the *positioning* (to the right or to the left) intrinsic to the pair: by this, we mean whether the two methylated loci in a pair occur (when ideally moving along the 5′-3′ direction) within the main strand first and within the COS then or, vice versa, first within the COS and then within the main strand. As an example, in M2-M1COS the CpG M2 (being the second CpG starting from 5′) is found within the gene strand *after* M1COS, and the CpG M1COS (being the *last* CpG starting from 5′ of antiparallel COS) is similarly found *later* than M2 on the opposed strand; in M2-M3COS, the CpG M3COS is found *earlier* on the opposed strand (being *before* CpG M2, starting from 5′ of antiparallel COS), and the CpG M2 (being the second CpG starting from 5′) is found *before* M3COS within the gene main strand. The first kind of situation was termed Rightward Positioning; vice versa, the second kind of situation was termed Leftward Positioning (see [1]).

**Table 1:** An extract of the pair-to-pair correlation matrix.

	M1-M1cos	M1-M2cos	M1-M3cos	M1-M5cos	M1-M6cos	M1-M7cos	…	M7-M7cos
**M2-M1cos**	\\	+0.4636	+0.3774	+0.4278	0.6889	−0.0523		−0.2370
**M2-M2cos**	+0.2124	\\	+0.3184	0.5682	+0.4794	−0.0571		−0.2445
**M2-M3cos**	+0.2200	+0.4576	\\	+0.5274	+0.4279	+0.0044		−0.1001
**M2-M5cos**	−0.1228	+0.1457	−0.0131	\\	+0.2716	−0.2325		−0.1976
**M2-M6cos**	+0.2130	+0.2837	+0.1057	+0.4869	\\	−0.1592		−0.1773
**M2-M7cos**	−0.2020	−0.0828	−0.0848	−0.1066	−0.0689	\\		\\
**…**								
**M6-M1cos**	\\	+0.3575	+0.5449	+0.1399	+0.1927	+0.0038		−0.0936
**M6-M2cos**	+0.3110	\\	+0.5574	+0.3782	+0.1553	+0.0748		−0.1241
**M6-M3cos**	+0.3196	+0.3741	\\	+0.1852	−0.0398	+0.1027		+0.0475
**M6-M5cos**	−0.0630	+0.1873	+0.1626	\\	+0.0818	−0.2309		−0.1470
**M6-M6cos**	+0.3092	+0.3204	+0.2377	+0.4519	\\	−0.2151		−0.1758
**M6-M7cos**	−0.0660	+0.0575	+0.2035	−0.1710	−0.2872	\\		\\
**…**								
**M7-M7cos**	−0.1733	−0.1338	−0.0291	−0.2518	−0.1616	\\		\
								
**Hint:**				cos5 + cos2	cos6 + cos1			

**Note:** \\ denotes the correlations where one member of the multiplied pair is the same; \ denotes the correlations where both members of the multiplied pair are the same.

**Figure 2:**
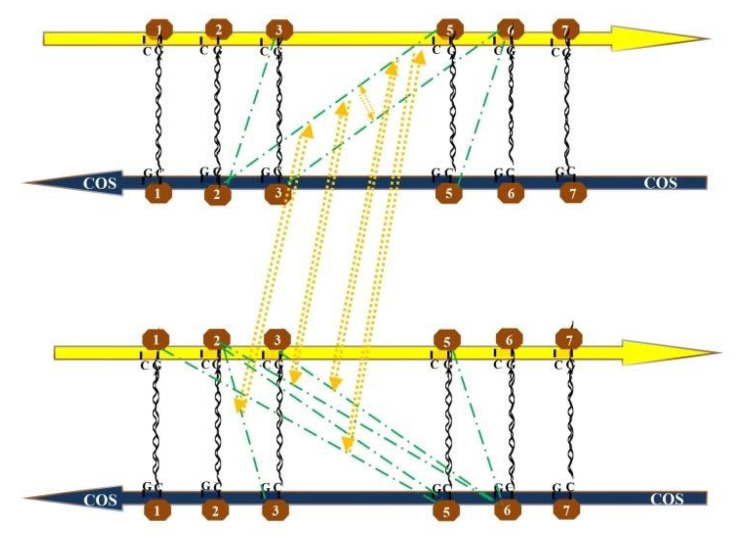
Graphic representation of significant recurrent interactions between all-specifically methylated CpG pairs, accounting for a tight interaction between cytosines placed one another at their left and giving clues for a rightward slip between the strands (see Figure 3). Each methylated pair is represented by the green point-line-point hatching, the correlations between pairs are drawn by the two-way dashed arrows in yellow.

In the end, only a fair number of highly correlated pairs emerged, which apparently organized themselves in recurrent loops. The most frequent loops are evident in Figure 1 and Figure 2, where each methylated pair is represented by the green point-line-point hatching, while the correlations between pairs are drawn by the two-way dashed arrows in yellow. To break down our findings, we followed the strategy of starting from pairs correlating with M6-M6COS:

- With reciprocal positioning to the right: M3-M2COS; M5-M2COS; M6-M5COS.
- With reciprocal positioning to the left: M2-M3COS; M2-M5COS; M2-M6COS; M1-M5COS; M3-M5COS; M3-M6COS; M5-M6COS.

We depict the looping network for right-positioned pairs suggesting a leftward slip (emerging from the M6-M6COS correlation table) in our first GRAPH: the strongest correlation with M6-M6COS is M3-M2COS (R = 0.7394); subsequently, we find that M3-M2COS links with M6-M5COS (R = 0.6266). Moreover, some main-strand de-methylated\COS-methylated pairs are known to be present (purple point-line-point hatching): namely, M3-M2COS correlates with D1-M6COS (R = 0.5616) and D1-M5COS (R = 0.5975) (see purple arrows in Figure 1).

In our second **GRAPH**, abundant loops are depicted for left-positioned pairs suggesting a rightward slip (Figure 2): another strong correlation with M6-M6COS is M2-M3COS (R = 0.6928). Notably, this is specular compared to the above; additionally, M6-M6COS points either to M3-M2COS or M2-M3COS, but not M2-M2COS (R = −0.5335). Subsequently, we find that M2-M3COS links with M5-M6COS (R = 0.7161) and M5-M2COS (R = 0.7877). Going to this latter, the full list of methylated pairs that correlate with M5-M2COS includes: M3-M6COS (R = 0.5798); M2-M6COS (R = 0.5804); M1-M5COS (R = 0.5785).

## 4. Discussion and Graphic Representations

To better understand the picture we got, it should be kept in mind that the M6-M6COS pair can be considered a starting point for two specular, non-overlapping sets of profiles: these are characterized by pair-to-pair correlations with pairs positioned to their right (e.g., M6-M5COS) as opposed to those with pairs positioned to their left (e.g., M5-M6COS). In the end, it will become clear that data suggest a “slip” between the two DNA strands, which we will term “leftward” or “rightward”, respectively. We cannot enter neither into the exact mechanism nor the architecture of this, so far; note however that DNA is known to be able to form secondary structures stabilized by cross-strand bonds among guanines [7,8,9]. Presently, we only are able to propose that the two sets of looping profiles may be consistent with either a rightward or a leftward sliding of the strands. 

As for *right-positioned pairs suggesting a leftward* slip, it can be inferred that the network of Figure 1 is based on a strict interaction between CpGs 2COS and 5COS with CpGs 3 and 6, respectively, through a “cascade” of correlations: such picture, by its topography suggesting the tight interaction of CpG 2 with CpG 1COS, is somewhat pointing towards a stable conformation, out of which a de-methylated CpG1 is emerging at the left of the highly pair-to-pair correlated trait. In other words, although this is just a suggestion, we propose that a tight, even physical interaction between CpGs 3 and 2COS as well as between CpGs 6 and 5COS (noteworthy, with a reciprocal positioning to the right) may somewhat stabilize a looping conformation whereby CpG1 can stay, in a de-methylated form, bysiding the loop itself.

As for *left-positioned pairs suggesting a rightward* slip, it is worthy of note that correlating pairs with M5-M2COS apparently include only combinations of CpGs 1 with CpGs 5COS and CpG 2 with CpG 6COS (but not CpG 1-6COS and CpG 2-5COS; Rs < 0.5). Moreover, similarly to the case seen above, these interactions are highly specific, as the correlation between CpGs 2-2COS and CpGs 5-6COS (permutation of the same elements) does not yield a significance (R = −0.3733). Once again, the tight interaction is between cytosines placed quite apart on strands, and not naturally nearby. In summary, specific pairs are appearing always correlated to given pairs, and only with similar reciprocal positioning (i.e., cytosines both to the right or both to the left).

### 4.1. Graphic Representation of Looping Flanks, with Simulation of Left and Right Seesaw 

The surprising finding was the great symmetry in a quite focused quantity of pair-to-pair correlations between the two motifs, in particular between CpGs 2 or 3 and 5 or 6 in both strands. When it comes to a physical or molecular substrate for such simple numerical and mathematical evidence, we realized that the whole picture would become somewhat easier to deal with, assuming that DNA bent to assume a looping conformation; whereby the two motifs would be close enough to allow cytosines 1\2\3 to interact with cytosines 5\6.

**Figure 3:**
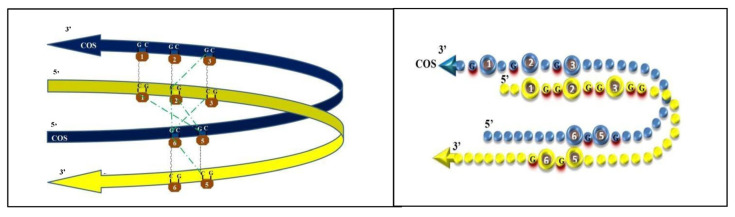
Graphic representation of looping strands (left panel); graphic representation by a pearl necklace model (right panel), with simulation of tight interaction between cytosines placed one another at their left, providing a cue for a rightward oscillation between DNA strands.

**Figure 4:**
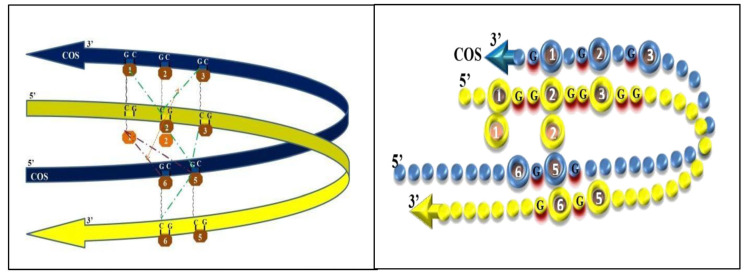
Graphic representation of looping strands (left panel); graphic representation by a pearl necklace model (right panel), with simulation of tight interaction between cytosines placed one another at their right, providing a cue for a leftward oscillation between DNA strands.

Once developed a graphical layout of the DNA folded in a looping fashion, the correlations of the linear graphs were reported, surprisingly assuming a simplified picture. CpGs 5 and 6 might take physical contact with CpGs 1-2-3, greatly easing the interpretation of their pair-to-pair correlations. The same green dotted lines of Figure 1 and Figure 2, when drawn on the looping graph, were clearly suggesting tight interactions between now very close cytosines. Such cytosines could become even closer, admitting that pair-to-pair correlations may suggest left and right slips, namely sliding movements of the COS relative to the main (gene) strand. A self-explaining drawing, with DNA represented as a necklace of pearls, has been prepared (Figure 3 and Figure 4).

In particular, in the oscillation dragged by cytosines lying to their left, pulling strands rightward (Figure 3), DNA presents the alignment as would be given by a pulling movement, driven by the external positions (i.e., correlations of M2-M3COS and M5-M6COS): the rightward slip of the main (gene) strand, sliding aside the COS strand, leads to the alignment of the sites M1 with M2COS. Probably, CpG1 is hence prevented from de-methylating, a notion that justifies the consistent finding of significance at M1-M5COS. The looping structure is possibly stabilized by the M3-M6COS link, which emerges clearly from the data. A global methylation may be hypothesized to occur and provide stability within the M1-M2COS/M5-M6COS space.

In the hypothesis of a four-strand local structure, the putative topography just illustrated has been termed “zone” of alignment.

In the oscillation dragged by cytosines lying to their right, pulling strands towards the left (Figure 4), the opposite alignment is produced. The pulling movement may again be driven by the external positions (now, correlations of M3-M2COS and M6-M5COS): the leftward slip of the main (gene) strand, sliding aside the COS strand, makes CpG M2 to eventually get aligned with CpG M1COS. At this point, we would find the CpG1 locus in a relatively external, free position to get de-methylated. Such a status may somewhat be confirmed by significant D1-M5COS and D1-M6COS pair-to-pair correlations being found. The apparent lack of cross-motif correlations favors the additional hypothesis that the whole looping may tend to be released.

In other words, diagonal pair-to-pair correlations may indicate forces that pull the opposite strands, which slip one relative to the other: in one case, CpG 2 with 1COS and CpG 6 with 5COS reach alignment (see Figure 3) while, in the other case, CpG 2 with 3COS and CpG 5 with 6COS do so (see Figure 4). The visual restitution through necklaces supports the idea that such methylation dynamics may alternatively lead the CpGs 1 and 6 to reach outer positions, allowing them to enter and to exit, like in a seesaw, the “zone” of alignment [4].

### 4.2. Some Hypotheses about Putative Secondary Structures 

The present work is currently limited to a graphic representation of the inferences drawn by data as discussed above, which obviously would require in-depth verification. The idea that DNA may form loops is quite common; additionally, we may propose that short motifs exist to allow the formation of local secondary structures [8,9,10,11]. Once such looping secondary structures are in place, it is becoming increasingly evident that epigenetic regulations occur [12,13,14,15]. 

DNA is associated with nucleosomes (i.e., a normal status for chromatin when transcriptionally active): one could actually argue that distant motifs may come close at each turn of DNA around a nucleosome. It is known that 147 bp make 1.7 turns in each nucleosome, so that portions of DNA may come into proximity every 86–87 bp. Since our motifs (CpG 1-2-3 and CpG 5-6) are far less distant (only 15 bp), we can exclude their putative proximity to be due to a turn around a nucleosome.

Being not so strange to imagine an alternative binding across the involved DNA chains, we have been searching for palindromic traits, and found partial overlaps (in bold between the main gene strand and the COS, written in the 5′-3′ direction):

TSS +711 gg**GC** ^1^ **GG**c ^2^ ggc ^3^ ggcttg**CC** ^4^ **GG**agactc ^5^ gc ^6^ gagct**CC** ^7^ **GC**a +748 AUG

   +748 t**GC**^7cos^**GG**agctc^6cos^gc^5cos^gagtct**CC**^4cos^**GG**caagcc^3cos^gcc^2cos^g**CC**^1cos^**GC**cc +711

A putative protein recognizing this consensus may bind to the main strand as well as to the COS, in that CpG 1 would be synonymous with CpG 7COS and CpG 7 of CpG 1COS. Anyway, it is also apparent that the CpG 7 motif is exactly complementary to the CpG 1 motif if going backward, and identical to the CpG 1COS motif on the inverted-direction COS (see [1], Figure 1). As such, the first CpG 1 “GCGG” motif on the main (gene) strand may alternatively bind to either identical sequence, one being its original COS as well as another found on the very same main strand, forty bp downwards, if this strand bends and comes back antiparallel after a loop. This second possibility would clearly stabilize intra-strand bonds if we admit that CpG 1 “GCGG” and CpG 7 “CGCC” (if coming backward, antiparallel) really bind. As the “GCGG” trait also characterizes CpGs 2 and 3, it may be proposed that the backward-antiparallel CpG 7 trait may choose whether to bind to either CpG 1, 2, or 3. The same is valid on the COS, of course. 

### 4.3. Some Hypotheses about Putative Strand Slipping 

The interaction of CpG 2 with either CpG 2COS or 1COS (see Table 1) is quite easy to imagine, as they are naturally close to one another. The interaction of CpG 1 with either CpG 5COS or 6COS is less easy to understand, unless we accept the loop bending of DNA. Yet, compared to the interaction of backward-antiparallel CpG 7 trait with CpGs 1 to 3, as proposed above, the putative interactions between CpGs 1 and\or 2 with CpGs 5COS and\or 6COS would happen on the double helix, before the opening of the two strains. As such, the DNA loop we proposed (with Figure 3 and Figure 4) may be hypothesized to serve as a checkpoint to allow or impede the DNA opening.

Interestingly, the CpG 2\2COS interaction (most obvious) comes along with the CpG 1\5COS one, thus giving a clue that a first step of the looping may be the placing of cytosine 5 aside cytosine 1. Then, the CpG 2\1COS interaction (less obvious) comes along with the CpG 1\6COS one (see Figure 4); thus, we are given a clue that the whole COS may slip to the right, relative to the main strand. Additionally, the whole COS may then be pulled along its 3′-5′ sense, and the same for the main strand, so that the two strands slip one relative to the other (see moving from Figure 4 to Figure 3). We termed “oscillation” the seesaw-like passing from Figure 4 to Figure 3 and vice versa, whereby reciprocal sliding of the two strands may even serve as a first step towards the opening of the double helix.

## 5. Conclusions

If our hypothesis is correct, the two alternative patterns of strand oscillations may either favor or contrast the continuation of DNA opening: indeed, one of the two oscillations would tend to rotate the double helix to make it unwind and open, whereas the other (opposed) oscillation would rotate the double helix to make it a supercoil, thus impeding further opening. In this perspective, our proposed approach (to study pair-to-pair correlations in methylation motifs) should be undertaken on data coming from other genes. A physical or chemical “direct” interaction between CpGs is just one possible prediction of our data; or, there may be “indirect” relationships via methyl binding proteins (MBP). When the CpGs become close due to a DNA loop, reciprocal links might be mediated by MBPs and then emerge through our matrix. Essentially, such a new approach may serve as a biomarker to be exploited in various medical conditions; yet, we should be keeping in mind a potential insight into the transcriptional status of such genes.

The above hypothesis cannot be proven at this stage by any evidence, since molecular and biophysical data would be needed which are far beyond the present purposes: this is a clear limitation of the present paper. Another limitation is that levels computed by traditional methylation analyses are not good estimators of the actual methylation probability at a given CpG. While future work is warranted, the present working hypothesis would be open to contribution by experts in the field. Why only specific pairs of main-strand and COS cytosines do correlate with only specific other pairs is an unexpected finding that shall mean something, in the end. In order to get a more precise account of the dynamic picture, similar studies with larger sample sizes should also apply a Bayesian correction. We are confident that our proposed approach, as well as the visual representation of the methylation dynamics, may represent a promising avenue.

## Data Availability

Data are stored on a PC in the office of the corresponding author and can be shared upon reasonable request.
